# Case report: Responses to the combination of gemcitabine with sirolimus in two patients with *TSC*-mutated sarcomas

**DOI:** 10.3389/fonc.2023.1046442

**Published:** 2023-02-09

**Authors:** Elise F. Nassif, Cissimol P. Joseph, Rossana Lazcano, Jocelyn T. Joseph, Prapassorn Thirasastr, Alexander J. Lazar, Neeta Somaiah

**Affiliations:** ^1^ Medical Oncology Department, Centre Léon Bérard, Lyon, France; ^2^ Department of Sarcoma Medical Oncology, The University of Texas MD Anderson Cancer Center, Houston, TX, United States; ^3^ Department of Translational Molecular Pathology, The University of Texas MD Anderson Cancer Center, Houston, TX, United States; ^4^ Division of Pathology and Laboratory Medicine, The University of Texas, MD Anderson Cancer Center, Houston, TX, United States; ^5^ Genomic Medicine, The University of Texas MD Anderson Cancer Center, Houston, TX, United States

**Keywords:** sarcoma, TSC, mTOR - mammalian target of rapamycin, gemcitabine, PEComa, nab-sirolimus

## Abstract

*TSC*-mutated sarcomas are rare molecular and histologic types of sarcoma. Due to the presence of their specific oncogenic driver mutation, these sarcomas are particularly sensitive to mTOR inhibitors. Recently, *nab*-sirolimus, an albumin-bound mTOR inhibitor, was approved by the Food and Drug Administration (FDA) for PEComas, which harbor a TSC mutation, and this drug remains the only FDA-approved systemic treatment for these tumors. We report on two cases of patients with *TSC*-mutated sarcomas who experienced significant responses to the combination of gemcitabine and sirolimus, after progression on prior gemcitabine-based chemotherapy and single agent mTOR inhibition with *nab*-sirolimus. Preclinical and clinical data support rationale for a synergistic effect of the combination. This combination may represent a valid therapeutic option after failure of *nab*-sirolimus in these patients, with no standard-of-care treatment options.

## 1 Introduction

Loss-of-function (LOF) mutations and deletions in Tuberous Sclerosis Complex (*TSC*) genes result in high activation of the mammalian target of rapamycin (mTOR) pathway and thus, tumors harboring these genomic alterations are particularly sensitive to mTOR inhibitors (1). In the sarcoma field, TSC alterations are rare but found mostly Perivascular Epithelioid Cell Tumors, known as PEComas (2), with a subgroup of these tumors localized in the uterus (3, 4).

Malignant PEComas are ultra-rare sarcomas (5) with an incidence ≤1/1,000,000 population every year. Due to the rarity of this disease, drug development is particularly challenging and unfortunately, conventional cytotoxic drugs have only modest activity on these tumors (6). Retrospective studies have consistently reported activity of single agent mTOR inhibitors in these sarcomas (7–9). Recently, the first prospective trial in this disease, the AMPECT study (10), demonstrated efficacy of *nab*-sirolimus, a novel and very potent mTOR inhibitor (11), in patients with advanced malignant PEComas, which led to the first Food and Drug Administration (FDA) approval in this disease in November 2021. In this landmark trial, the objective response rate was 39% and the median progression-free survival was 10.6 months. Although these results are encouraging, there are currently no other approved systemic drug for patients after progression on *nab*-sirolimus.

Gemcitabine is a commonly prescribed cytotoxic drug in the sarcoma field (12, 13). For malignant PEComas, retrospective data demonstrated that gemcitabine-based chemotherapy regimens have a 20% objective response rate and a median progression-free survival of 3.4 months (6).

A phase 1 study across several cancer types demonstrated safety of the combination of sirolimus 5mg daily, an oral mTOR inhibitor, with gemcitabine 800mg/m^2^ on days 1 and 8 (14). As part of this phase 1 study, *in vitro* and *in vivo* studies suggested that gemcitabine induces mTOR pathway hyperactivation, which was reversed by sirolimus.

We report on two cases of patients with *TSC*-mutated sarcomas who experienced significant and prolonged responses to the combination treatment of gemcitabine and sirolimus, after progression on gemcitabine-docetaxel and nab-sirolimus treatments.

Both patients provided informed consent for publication of their cases.

## 2 Case 1

**Figure 1:**
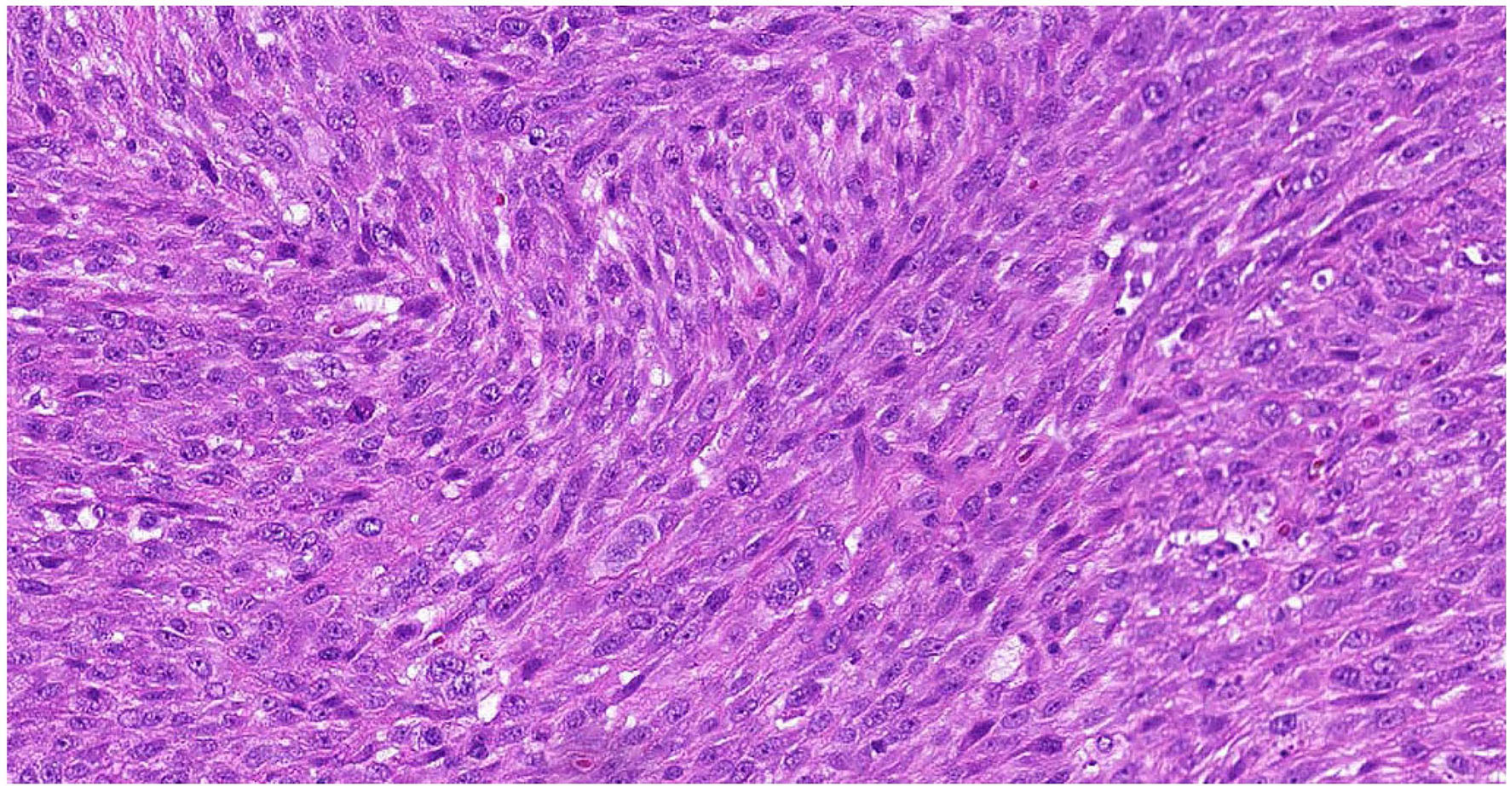
Representative Hematoxylin & Eosin Slide of Outside Referral Biopsy from Patient 1. Sample collected in February 2020, pre-treatment, image is at 10X magnification.

A 52-year-old female with a past medical history of uterine fibroids resected in 2013, hyperglycemia, and hypertension, presented with pelvic pain and menorrhagia in January 2020, at which time a 4.4cm intrauterine mass was found on ultrasound. In February 2020, the patient underwent an exploratory laparotomy with total abdominal hysterectomy, radical pelvic dissection, bilateral salpingo-oophorectomy, and bilateral pelvic and para aortic lymph node dissection. Pathologic evaluation of the uterine mass revealed a large (>15cm) high-grade myxoid pleomorphic spindle and epithelioid sarcoma **(** **Figure 1** **)**, positive metastatic lymph nodes, and tumor present at the resection margins. Post-operative computed tomography (CT) revealed bilateral pulmonary and mesenteric nodules, and a residual pelvic mass. She came to see us at this time.

Systemic treatment was initiated on April 1^st^, 2020 with gemcitabine 900mg/m^2^ (day 1 and day 8) and docetaxel 75mg/m^2^ (day 8) every 21 days, with initial treatment response in thoracic, mesenteric, and pelvic disease. After eleven cycles of this regimen (tolerated well with dose and schedule adjustments), disease progression was noted in December 2020. The systemic treatment was changed to doxorubicin (75mg/m^2^) and dacarbazine (750mg/m^2^) every 21 days. There was a rapid disease progression on CT-imaging after three cycles with this treatment regimen.

At this time the molecular profiling of the tumor by targeted panel (in house next-generation sequencing) was available and revealed LOF mutations in *TSC2*, *TP53*, and *ATRX*, and a copy-number loss of *RB1*. The patient was subsequently offered targeted treatment for the oncogenic *TSC2* LOF mutation and participated in an early phase trial of *nab*-sirolimus 100mg/m^2^ on day 1 and day 8 every 21 days, with initial response to treatment after two cycles, followed by disease progression after six cycles (NCT03817515). The treatment was overall well tolerated, with grade 1 neutropenia, grade 2 hypertriglyceridemia, and grade 1 maculo-papular rash. The patient was subsequently offered and participated in an early phase trial targeting the ATRX LOF mutation, through a combination of a PARP inhibitor and an ATR inhibitor. However, the patient experienced severe abdominal pain with marked abdominal disease progression requiring hospitalization after just one cycle of this therapy and was considering hospice.

**Figure 2:**
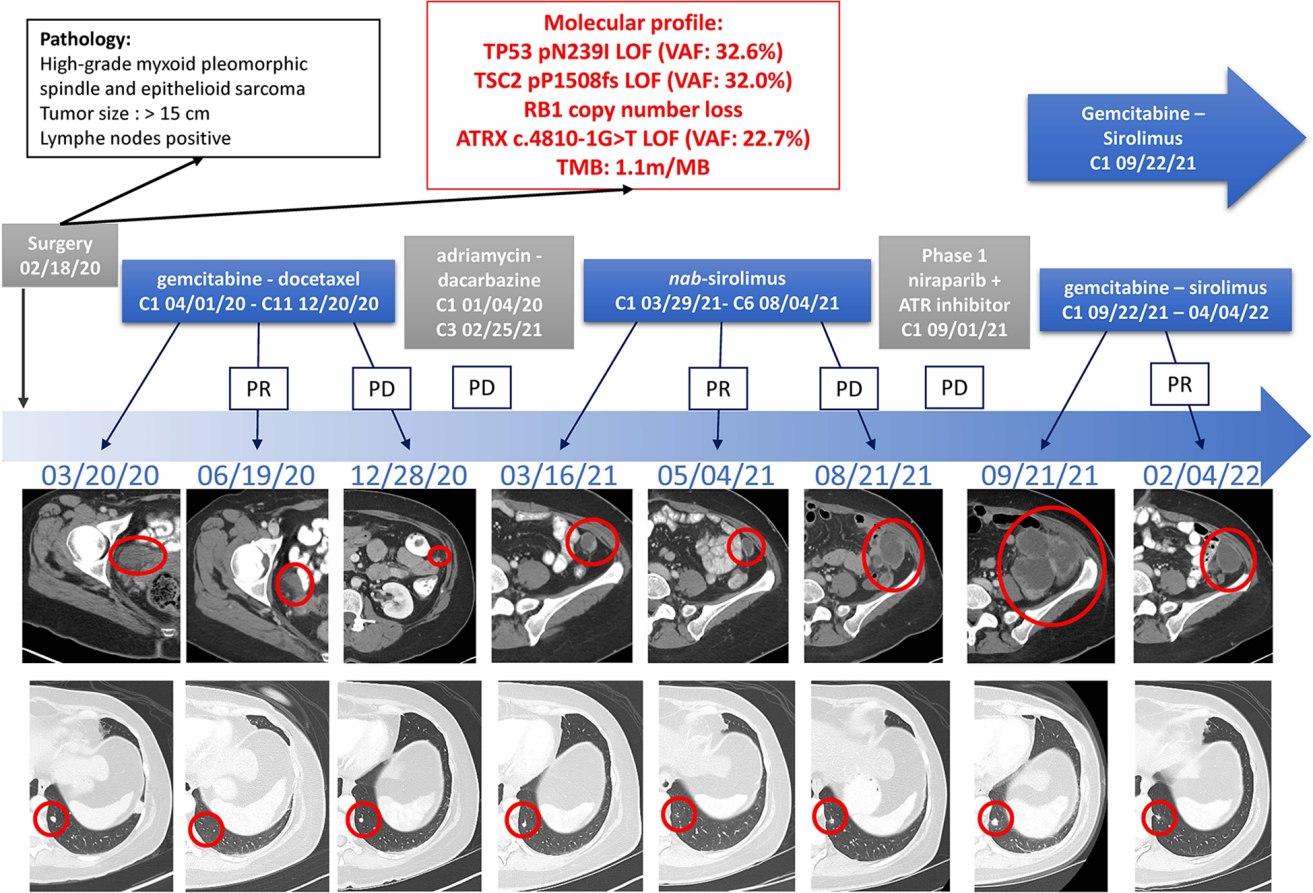
Timeline of Treatments of Patient 1. Treatment lines are displayed chronologically, with imaging assessments at baseline, at time of best response to treatment, and at time of end of treatment for each line of treatment. Initial pathology diagnostic and molecular profiles are displayed. *IHC, immunohistochemistry; LOF, loss-of-function; MSI, microsatellite instable; PD, Progressive Disease; PR, Partial Response; TMB, tumor mutational burden; VAF, variant allele fraction*.

Based on the prior disease response to gemcitabine and mTOR inhibition, the patient started a combination regimen of gemcitabine 800mg/m^2^ on day 1 and day 8 with sirolimus 5mg daily every three weeks on September 29^th^, 2021. The patient experienced rapid symptom improvement and discontinued morphine two weeks after the start of treatment. CT-imaging after two cycles showed marked tumor regression. After eight cycles of treatment, the disease displayed mixed response, whereby some of the responding sites of disease remained stable while others started to display mild increase. The drug doses were increased on March 21^st^, 2022 to 900m/m^2^ of gemcitabine and 7mg of sirolimus, subsequently dropped back to 5 mg due to toxicity and plasma drug levels. After ten cycles on this therapy, a left paracolic implant eroded into the descending colon, resulting in a fistula, and treatment had to be held since April 4^th^, 2022. Her performance status remains better than 8 months ago, before starting treatment (**Figure 2**).

## 3 Case 2

**Figure 3:**
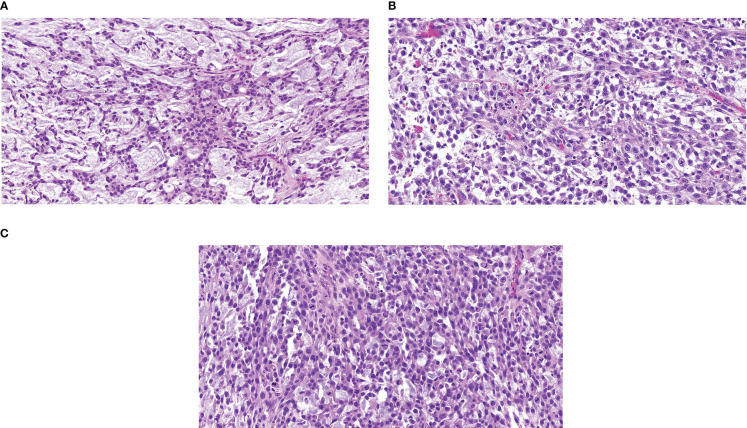
Representative Hematoxylin & Eosin Slide of Outside Referral Biopsy from Patient 2 Samples collected in December 2020 **(A)**, January 2021 **(B)**, and March 2021 **(C)**, all pre-treatment, image is at 10X magnification.

An 82-year-old female with a past medical history of breast cancer in 1999 and 2002, hypertension, and hypothyroidism presented in October 2020 with a left lower extremity mass following a fall. A hematoma was suspected and thus, she underwent incomplete resection revealing a 5.5cm high-grade myxofibrosarcoma of the thigh with 23 mitosis in 10 high power field and 20% necrosis on December 4^th^, 2020 **(** **Figure 3** **)**. The patient underwent repeat incomplete resection in January and March 2021 at an outside institution. CT-imaging revealed a pelvic mass, a left posterior thigh residual nodule, and bilateral sub-centimeter lung nodules, and the patient started a combination regimen of gemcitabine 675mg/m^2^ (day 1 and day8) and docetaxel 75mg/m^2^ (day 8) every 21 days on May 25^th^ 2021, which was held after first cycle due to increasing rectal discomfort from the enlarging pelvic mass requiring a diverting colostomy. She presented to us after this.

The molecular profile by targeted gene panel (in-house next-generation sequencing) revealed LOF mutations in *TSC1*, *TP53*, and *ATRX*, and a copy-number loss of *CDKN2A* and *CDKN2B*. The patient was offered participation in the same *nab*-sirolimus trial as our first reported patient. After two cycles, CT-imaging showed a mild increase of the pelvic mass and residual left posterior thigh nodule (stable by RECIST 1.1), and treatment was continued for two more cycles after discussion with the patient and study coordinator. Unfortunately, CT-imaging after these additional two cycles confirmed disease progression (by RECIST 1.1) and the patient discontinued *nab*-sirolimus. The tolerance of *nab*-sirolimus was marked by grade 3 neutropenia, grade 2 fatigue and pain, and grade 1 oral mucositis.

From November 11^th^ to December 2^nd^, 2021, the pelvic mass and left thigh nodule were treated with radiation therapy of 45Gy. The patient subsequently participated in an early phase trial of a doxorubicin prodrug starting on January 6^th^, 2022. However, after two cycles on trial, there was marked increase in the previously noted sub-centimeter lung nodules with new lung metastases.

**Figure 4:**
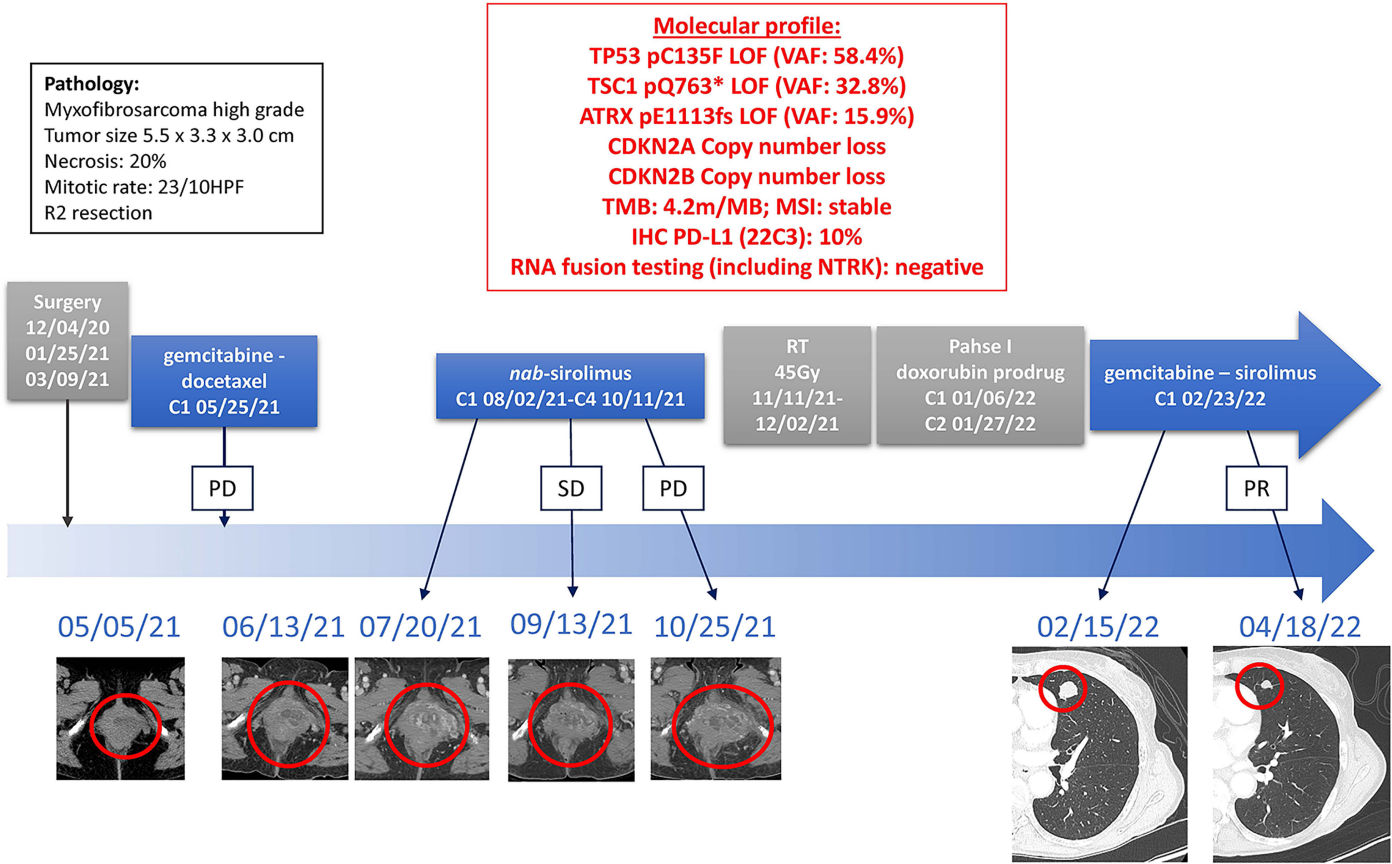
Timeline of Treatments of Patient 2. Treatment lines are displayed chronologically, with imaging assessments at baseline, at time of best response to treatment, and at time of end of treatment for each line of treatment. Initial pathology diagnostic and molecular profiles are displayed. *HPF, high power field; IHC, immunohistochemistry; LOF, loss-of-function; MSI, microsatellite instable; PD, Progressive Disease; PR, Partial Response; SD, Stable Disease; TMB, tumor mutational burden; VAF, variant allele fraction*.

Due to the exceptional response of our first reported patient, the patient was offered treatment with a combination of gemcitabine 675mg/m^2^ every 2 weeks with sirolimus 5mg daily. After two cycles of this combination regimen, CT-imaging displayed marked regression of lung nodules and treatment is ongoing. Toxicities noted on-treatment include grade 3 neutropenia and anemia, grade 2 fatigue, and grade 1 pelvic pain residual from radiation treatment **(** **Figure 4** **)**.

## 4 Discussion

In our two similar cases of uterine *TSC*-mutated metastatic sarcomas, both patients had an exceptional response to the combination of gemcitabine with sirolimus, after failure of gemcitabine-docetaxel and of *nab*-sirolimus.

Both patients had similar molecular profiles with *TSC* LOF mutations, *TP53* LOF mutations, *ATRX* LOF mutations, and cell-cycle pathway copy-number losses, which could be consistent with the diagnosis of uterine PEComas (4), although other sarcoma types have also been reported with these genomic profiles. Clinically, both patients experienced disease progression on gemcitabine-docetaxel combination and on *nab*-sirolimus, followed by an exceptional response to the combination of gemcitabine and sirolimus. However, our second patient, whose tumor harbored a *TSC1* mutation, had no clinical benefit neither from gemcitabine-docetaxel nor from *nab*-sirolimus, whereas our first patient, whose tumor harbored a *TSC2* mutation, had an initial response to both lines of treatment. In the AMPECT trial of *nab*-sirolimus, *TSC2* LOF mutations were significantly associated with improved response to *nab*-sirolimus, but not *TSC1* LOF mutations (10), which may explain this discrepancy between the apparent sensitivity to gemcitabine-docetaxel and *nab*-sirolimus between our two patients. However, the combination of gemcitabine and sirolimus was effective even in our patient with a *TSC1*-mutated sarcoma.

Several pre-clinical models have shown a synergistic effect of combining mTOR inhibitors with gemcitabine (15–22). Mechanistically, gemcitabine has shown to induce hyperactivation of the AKT/PI3K/mTOR pathway which is targeted and reversed by mTOR inhibitors (14, 18, 20). Clinically, the combination of gemcitabine with mTOR inhibitors has shown promising activity in prospective trials in osteosarcoma (23), in advanced soft-tissue sarcomas (24, 25), and other solid tumors (14, 26), without prior molecular screening for *TSC* mutations.

In *TSC*-mutated sarcomas, with no standard-of-care treatment after failure of *nab*-sirolimus, the combination of gemcitabine with sirolimus may be an effective treatment option, which warrants further investigation.

## Data availability statement

The original contributions presented in the study are included in the article/supplementary material. Further inquiries can be directed to the corresponding author.

## Ethics statement

Ethical review and approval was not required for the study on human participants in accordance with the local legislation and institutional requirements. The patients/participants provided their written informed consent to participate in this study. Written informed consent was obtained from the individual(s) for the publication of any potentially identifiable images or data included in this article.

## Author contributions

EN: data curation, visualization, writing – original draft preparation, writing – review and editing. CJ: investigation, writing – review and editing. RL: visualization. JJ: writing – review and editing. PT: writing – review and editing. AL: visualization. NS: investigation, methodology, supervision, visualization, writing – original draft preparation, writing – review and editing. All authors contributed to the article and approved the submitted version.
